# Identification and Characterization of Tropomyosin 3 Associated with Granulin-Epithelin Precursor in Human Hepatocellular Carcinoma

**DOI:** 10.1371/journal.pone.0040324

**Published:** 2012-07-06

**Authors:** Ching Yan Lam, Chi Wai Yip, Terence C. W. Poon, Christine K. C. Cheng, Eddy W. Y. Ng, Nicholas C. L. Wong, Phyllis F. Y. Cheung, Paul B. S. Lai, Irene O. L. Ng, Sheung Tat Fan, Siu Tim Cheung

**Affiliations:** 1 Department of Surgery, The University of Hong Kong, Hong Kong; 2 Centre for Cancer Research, The University of Hong Kong, Hong Kong; 3 State Key Laboratory for Liver Research, The University of Hong Kong, Hong Kong; 4 Li Ka Shing Institute of Health Sciences, Department of Paediatrics, The Chinese University of Hong Kong, Hong Kong; 5 Department of Surgery, The Chinese University of Hong Kong, Hong Kong; 6 Department of Pathology, The University of Hong Kong, Hong Kong; National Cancer Institute, United States of America

## Abstract

**Background and Aim:**

Granulin-epithelin precursor (GEP) has previously been reported to control cancer growth, invasion, chemo-resistance, and served as novel therapeutic target for cancer treatment. However, the nature and characteristics of GEP interacting partner remain unclear. The present study aims to identify and characterize the novel predominant interacting partner of GEP using co-immunoprecipitation and mass spectrometry.

**Methods and Results:**

Specific anti-GEP monoclonal antibody was used to capture GEP and its interacting partner from the protein extract of the liver cancer cells Hep3B. The precipitated proteins were analyzed by SDS-PAGE, followed by mass spectrometry and the protein identity was demonstrated to be tropomyosin 3 (TPM3). The interaction has been validated in additional cell models using anti-TPM3 antibody and immunoblot to confirm GEP as the interacting partner. GEP and TPM3 expressions were then examined by real-time quantitative RT-PCR in clinical samples, and their transcript levels were significantly correlated. Elevated TPM3 levels were observed in liver cancer compared with the adjacent non-tumorous liver, and patients with elevated TPM3 levels were shown to have poor recurrence-free survival. Protein expression of GEP and TPM3 was observed only in the cytoplasm of liver cancer cells by immunohistochemical staining.

**Conclusions:**

TPM3 is an interacting partner of GEP and may play an important role in hepatocarcinogenesis.

## Introduction

Hepatocellular carcinoma (HCC) is a malignant neoplasm of hepatocytes and it accounts for more than 80% of primary liver cancers [1]–[2]. HCC is a major global health problem. It shows significant regional variations with a very high incidence rate in Asia and Sub-Saharan Africa compared with the Western countries, where there is also increasing incidence. In Hong Kong, HCC is the fourth most common cancer and the mortality rate ranks the third. The main etiological factors for HCC include alcoholic cirrhosis, infection of hepatitis viruses B and C, chronic exposure to aflatoxin B1 and haemochromatosis. In addition, alpha-1-antitrypsin deficiency and Wilson’s disease are also potential risk factors for HCC development. Although the curative treatment for HCC is surgical resection or liver transplantation, only a minority of HCCs are amenable to surgery as symptoms attributable to HCC usually develop in the late stages of the disease. Besides, most of the HCC patients have advanced cirrhosis which leads to insufficient hepatic remnant and normal liver function after liver resection and hence, surgical resection is not applicable for many patients. Another concern is the high recurrence rate after surgical resection. Fifty to eighty percent of patients suffer disease recurrence, which could be intrahepatic metastasis or multicentric occurrence, within five years after resection. Chemotherapy is an alternative treatment of HCCs. However, only marginal efficacy has been observed and severe side effects are hurdle to the feasibility of chemotherapy [1]–[2].

Several important intracellular signaling pathways including the mitogen-activated protein kinases comprising the ERK, JNK and p38 have been recognized to be involved in hepatocarcinogenesis [3]. In addition, several growth factors and angiogenic factors such as EGF and VEGF have been suggested to contribute to HCC [3]. However, the molecular pathogenesis of HCC has not been well characterized yet. It is a major global health problem, and the prognosis is dismal. The need for better understanding of the cellular and molecular mechanisms of the disease is obvious and crucial to disease prevention and management. Recently, the advanced cDNA microarray technology has greatly facilitated the genome-wide expression profiling in many complex diseases such as cancers. Understanding the gene expression profiles in HCC may provide new insights in identifying novel candidate biomarkers for early diagnosis and discovery of therapeutic targets for cancer treatment.

**Figure 1:**
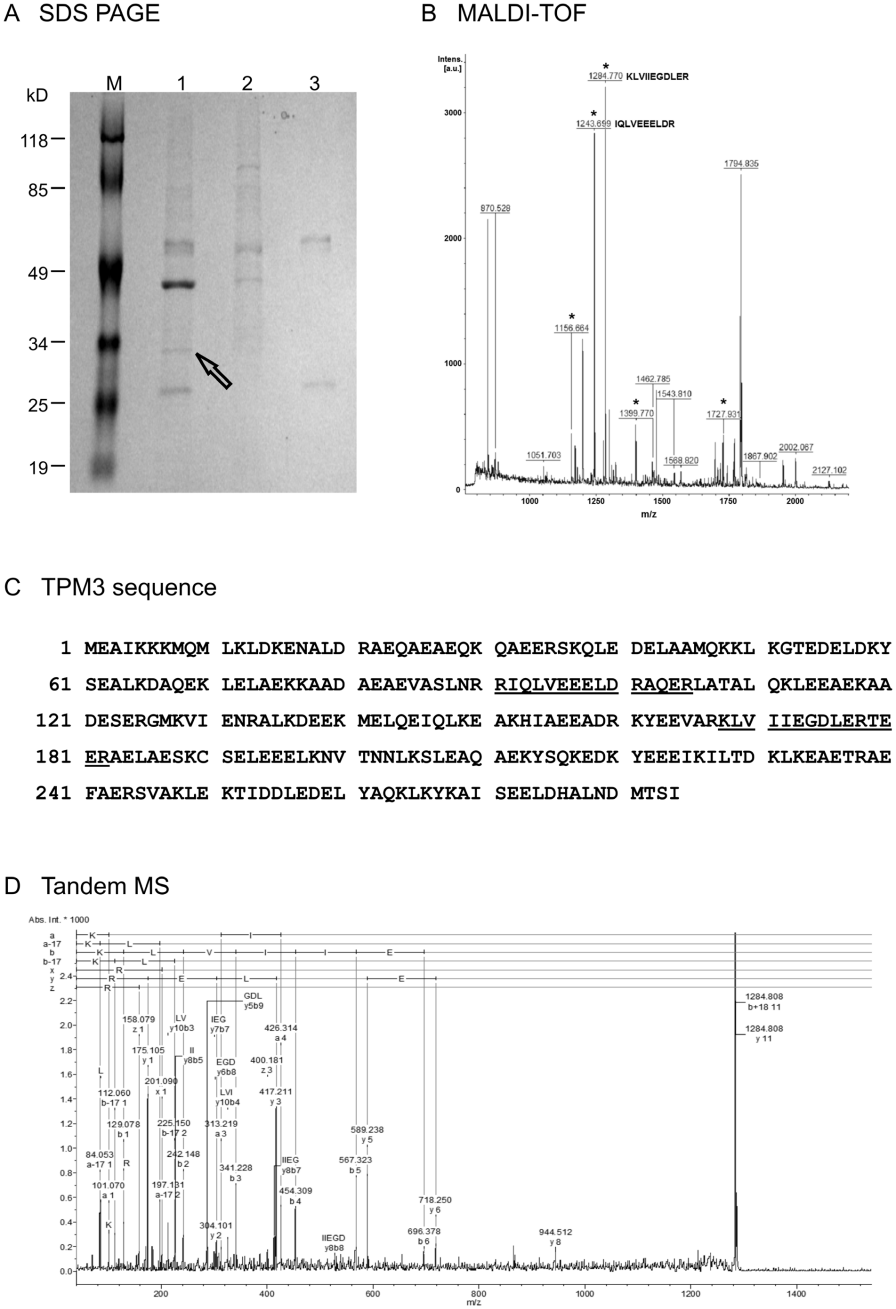
Identification of TPM3 as the predominant binding partner of GEP. (A) SDS-PAGE analysis of co-immunoprecipitation reactions using total protein lysate from liver cancer cells Hep3B and monoclonal anti-GEP antibody. M: Protein marker (Fermentus prestained protein marker); Lane 1: Immunoprecipitation product of protein lysate and anti-GEP antibody; Lane 2: Protein lysate alone; Lane 3: Anti-GEP antibody alone. The protein band indicated by the arrow was excised from the gel and the protein identity was examined by MS. (B) MALDI-TOF/TOF-MS analysis of the tryptic peptides. The peptides matched to TPM3 were asterisked. (C) Protein sequence of TPM3 was shown, and matched peptides were underlined. (D) An annotated representative tandem MS mass spectrum of a tryptic peptide (M/Z 1284.8) with the amino acid sequence identified as KLVIIEGDLER.

**Table 1:** Summary of the peptide mass fingerprinting (PMF) and MS/MS ion search results of the predominant protein-protein interaction partner.

Swiss-Prot AC	Protein name	Observed apparent MW (kDa)	Calculated MW (kDa)	PMF	MS/MS ion search^a^			
				Expectation value	Observed m/z	Calculated m/z	Expectation value (Mowse score)	Peptide sequence
P06753	Tropomyosin alpha-3 chain (TPM3)	34	32.8	3.9E−8	1243.70 1284.79	1243.65 1284.74	0.0061 (35) 0.00017 (46)	IQLVEEELDR KLVIIEGDLER

AC, accession code; PMF, peptide mass fingerprinting; obs, observed; calc, calculated; MW, molecular weight;

a the search results of 2 best matched peptides were provided.

Our earlier cDNA microarray study revealed differential gene expression patterns in HCC and non-tumor liver tissues [4]–[5]. Granulin-epithelin precursor (GEP) expression was observed in over 70% of HCC [6]. Functional studies revealed that GEP controlled cancer cells proliferation, invasion and chemo-resistance [6]–[7]. We therefore investigated the potential of GEP as a therapeutic target. Anti-GEP monoclonal antibodies were developed and demonstrated to be able to inhibit the growth of hepatoma cells but no effect on normal liver cells [8]. In nude mice model transplanted with human HCC, dose-dependent inhibitory effect was demonstrated with the anti-GEP monoclonal antibodies, providing evidences that GEP is a therapeutic target for HCC treatment [8]. GEP expression has also been reported in a number of aggressive tumors, involved in various biological processes including wound healing, murine fetal development, and mutation associated with frontotemporal lobar dementia [9]. GEP has been reported to interact with Tat proteins of Human Immunodeficiency Virus (HIV), with COMP and TNF receptors in chondrocyte [10]–[12]. Nonetheless, the GEP interacting partners/receptors have yet to be identified in cancer cells [13]–[14].

**Figure 2:**
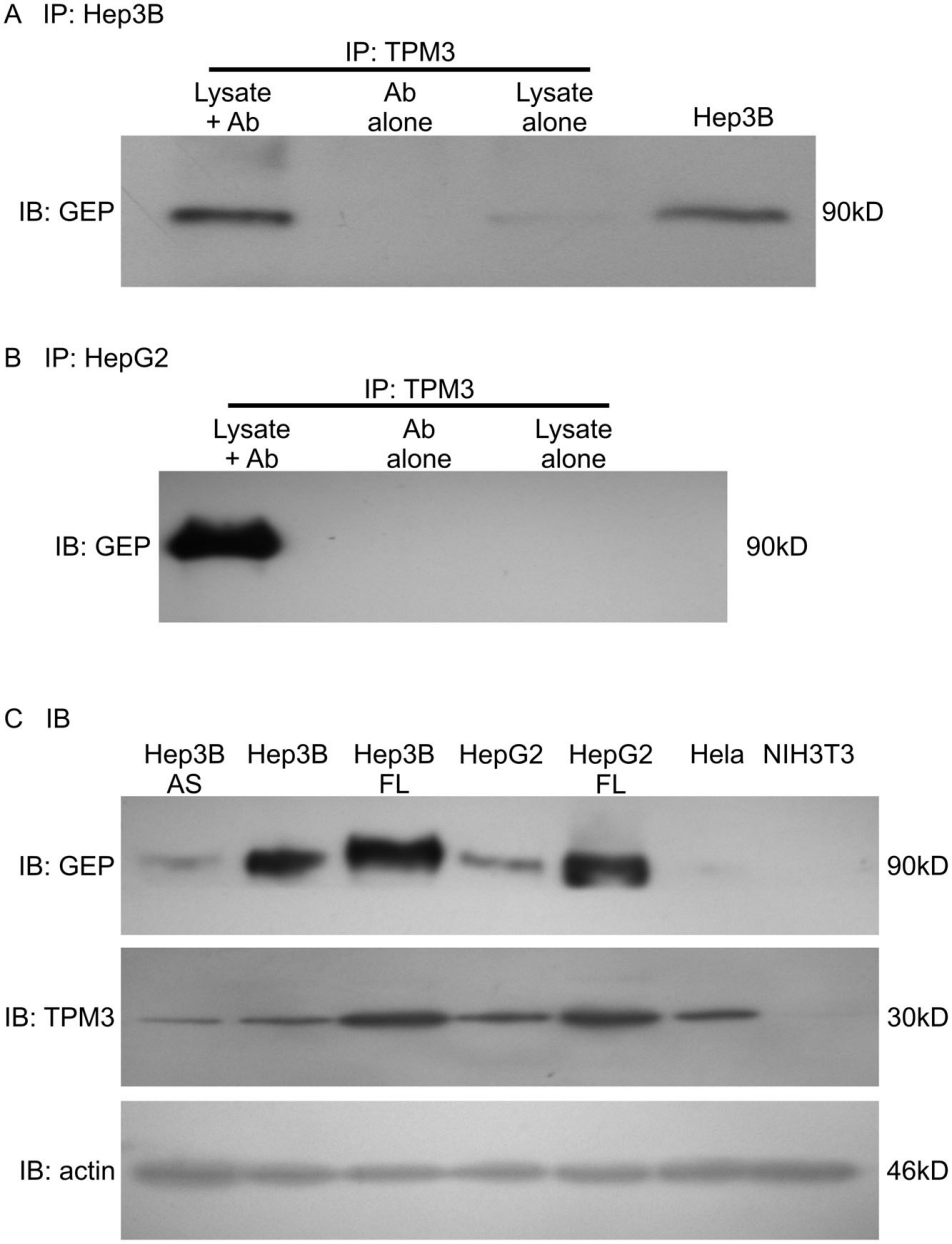
Association of TPM3 and GEP. (A–B) Protein-protein interaction of TPM3 and GEP was validated by immunoprecipitation using anti-TPM3 antibody, followed with immunoblot detection using anti-GEP antibody in different cell lines (A) Hep3B and (B) HepG2 cells. (C) Positive correlation of GEP and TPM3 on protein level. AS: Cells suppressed for GEP by transfection with anti-sense GEP fragment; FL: Cells overexpressed for GEP by transfection with full-length (FL) GEP cDNA construct. Cells with overexpression of GEP showed elevated TPM3 levels, while suppression of GEP showed decreased TPM3.

To further understand the GEP signaling mechanism, the present study aims to identify its novel predominant interacting partners. Proteins that interact with GEP were examined using co-immunoprecipitation and mass-spectrometry. The GEP interacting protein had been further examined in additional cell lines and clinical samples using western blot, immunohistochemistry and real-time quantitative PCR.

**Figure 3:**
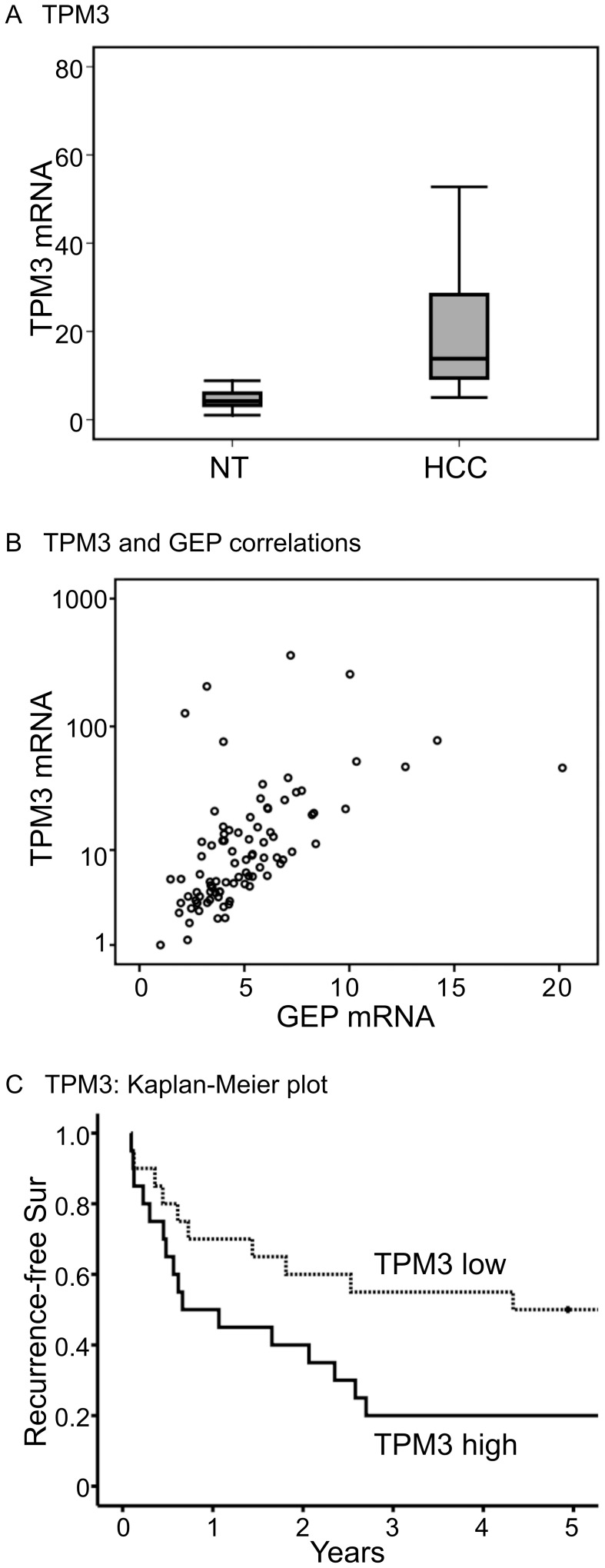
Over-expression of TPM3 and GEP mRNA levels in liver cancer. (A) Elevated expressions of TPM3 were observed in liver cancer (hepatocellular carcinoma, HCC) compared with the paralleled adjacent non-tumor liver tissues (*P*  = 0.001, respectively). (B) Transcript levels of GEP and TPM3 were significantly correlated (Spearman’s ρ correlation coefficient  = 0.658, *P*<0.001). (C) Kaplan-Meier recurrence-free survival plot according to TPM3 levels (log-rank test, *P*  = 0.0496). Patients in the TPM3-high group showed poor recurrence-free survival compared to TPM3-low group (median recurrence-free survivals 8.0 and 52.0 months, respectively).

## Materials and Methods

### Antibodies

Anti-GEP monoclonal antibody raised against the GEP carboxyl-terminus was used for immunoprecipitation [8]. Antibodies for TPM3 (polyclonal antibodies raised against the low molecular weight isoform 2, Sigma-Aldrich, St. Louis, MO), anti-mouse IgG and anti-rabbit IgG secondary antibodies (Dako, Carpinteria, CA) were purchased.

**Figure 4:**
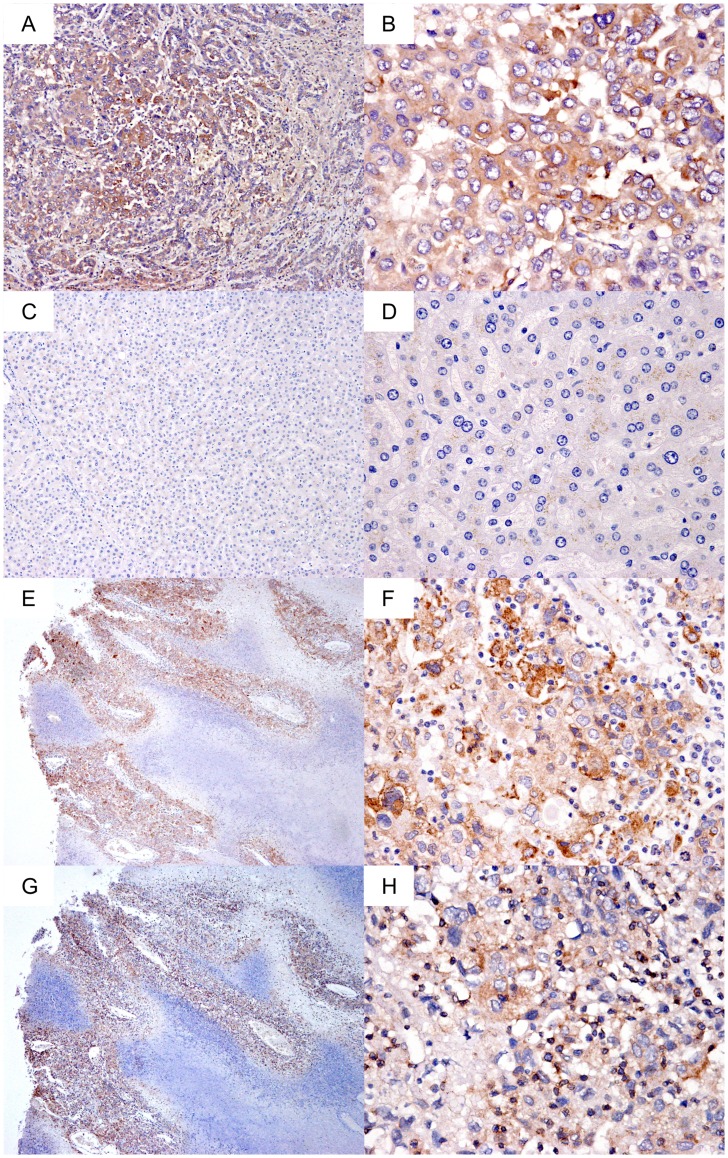
Immunohistochemistry analysis of TPM3 and GEP on clinical specimens. (A–D) Elevated TPM3 protein in liver cancer. TPM3 was observed in the cytoplasm of liver cancer cells (A and B) but rarely in the adjacent non-tumor liver tissue (C and D). (E–H) GEP and TPM3 co-localization in liver cancer. Similar pattern of protein expressions were observed in GEP protein (E and F) and TPM3 protein (G and H). The protein signal was visualized as brown. Magnification×100 in A, C, E and G; ×400 in B, D, F and H.

### Cell Lines

Two human liver cancer cell lines, Hep3B and HepG2 (American Type Culture Collection, ATCC, Manassas, VA), were used in the immunoprecipitation experiments. Hep3B was derived from an 8 years old juvenile patient, whereas HepG2 was derived from a 15 years old adolescent patient. The two cell lines were grown in AMEM medium containing 10% fetal bovine serum (FBS) and L-glutamine supplement at 37°C in 5% CO_2_. GEP full-length cDNA and anti-sense fragment construct were transfected into the liver cancer cells to obtain stable transfectants for GEP overexpression and GEP suppression, respectively. Stable clones were selected by G418 as described previously [6]. Another two cell lines Hela and NIH3T3 (ATCC) were used as references in the immunoblotting experiments. HeLa was adenocarcinoma cells derived from the cervix of a 31 years old patient, whereas NIH3T3 was mouse fibroblast cell line derived from the mouse embryo.

### Clinical Samples

The studpy protocol was approved by the Institutional Review Board of the University of Hong Kong/Hospital Authority Hong Kong West Cluster (HKU/HA HKW IRB). Patients underwent curative partial hepatectomy for HCC at Queen Mary Hospital, Hong Kong, were recruited with written inform consent to the study. Total RNA was extracted from snap frozen tissue specimens for mRNA expression study using real-time quantitative PCR, whereas formalin-fixed paraffin-embedded tissues were used for histological and immunohistochemical studies.

### Coimmuniprecipitation

Total cell lysates were obtained using NET buffer (50 mM Tris-HCl pH7.5, 15 mM EDTA, 100 mM NaCl, 0.1% Triton X-100) in the presence of complete protease inhibitor (Roche) and 1 mM phenylmethylsulphonyl fluoride (USB, Cleveland, OH). Briefly, cells were first washed by PBS and cell pellet was collected by trypsinization. The cell pellet was washed using PBS and then NET was added to resuspend the cells. The lysate was then incubated on ice for 30 minutes and followed by centrifugation at 14,000 x g for 20 minutes at 4°C. The supernatant was collected and the concentration of the total protein was determined using the BioRad DC Protein Assay Kit (BioRad, Philadelphia, PA). A total of 400 µg extracted protein lysate was used for each co-immunoprecipitation reaction. The monoclonal anti-GEP antibody which is specific to human GEP was used to coimmunoprecipitate GEP and its binding partners. Two control reactions, antibody alone and protein lysate alone, were also included as reference to the non-specific binding of unrelated proteins with the protein G-Sepharose. Two microgram of monoclonal anti-GEP antibody was incubated with 400 µg total protein lysate at 4°C with rotation overnight. For each reaction, 100 µl of protein G-Sepharose beads (Amersham Biosciences, Piscataway, NJ) were washed with 500 µl NET buffer for 3 times. Washed protein G-Sepharose beads were incubated with the antibody-protein complexes at 4°C with rotation for 1 hour. After incubation, the complexes were briefly centrifuged and supernatant was discarded. The beads were washed with 500 µl ice-cold NET buffer for 5 times to wash any unbounded proteins.

### Identification of the Major Interacting Partner by SDS-PAGE

The coimmunoprecipition product was then analysed by typical one-dimensional SDS-PAGE. The immunocomplex-bound protein G-Sepharose beads were resuspended using 2×protein buffer (4×Tris.CL/SDS, pH6.8, glycerol, bromophenol blue and β-mercaptoethanol). Proteins were denatured at 95°C for 5 minutes. The supernatant containing the proteins were separated under denaturing condition on 10% SDS-PAGE gel and followed by SimplyBlue (Invitrogen, Carlsbad, CA) staining which is a mass-spectrometry compatible coomassie blue stain. The major differential band observed in the co-immunoprecipitation reaction but absent in the two control reactions (antibody alone and protein lysate alone) were excised from the gel and further analyzed by mass-spectrometry.

### Protein Identification by Mass Spectrometry

The differential protein band was excised from stained gel, and in-gel trypsin digestion was performed as previously described [15]. The gel pieces were destained, reduced with 1.75% DTT, alkylated with 350 mM iodoacetamide (IAA), and digested with modified porcine trypsin overnight (sequencing grade, Promega, Madison, WI). The tryptic peptides were harvested, cleaned up with C18 ZipTips (Millipore Corp., Billerica, MA), and subjected to MALDI-TOF/TOF MS (Ultraflex-III, Bruker Daltonics, Bremen, Germany) with α-cyano 4-hydroxy cinnamic acid as the matrix. The MS and MS/MS spectra were automatically processed with the FlexAnalysis program (version 3.0, Bruker Daltonics) with the default parameters. The MS spectrum data were searched via the MASCOT search engine to obtain the protein identity by undertaking the peptide mass fingerprinting (PMF) approach and the MS/MS ion search approach. For the search parameters, 1 missed cleavage in trypsin digestion was allowed; partial oxidation of methionine, phosphorylation of serine/threonine/tyrosine, and iodoacetamide modification of cysteine residues were selected. The error tolerance values of the parent peptides and the MS/MS ion masses were 50 ppm and 0.1 Da, respectively. For a gel spot, an identification result was considered valid when both PMF and MS/MS ion search identified the same protein as the statistically significant hit (expectation value <0.05) from the Swiss-Prot database, and MS/MS ion search identified at least 2 tryptic peptides with sequences from the same protein as the statistically significant hits.

### Western Blotting

For protein expression on cell lines, aliquot of 20 µg total protein lysate were separated in 10% SDS-PAGE gel [6], [8]. Proteins were then electro-transferred onto polyvinylidene fluoride membranes and subsequently incubated overnight at 4°C with primary antibodies against GEP or TPM3. Detection was performed by horseradish peroxidase-labelled secondary antibodies with enhanced chemiluminescence (AP Biotech, Chalfont St. Giles, UK).

### Real-time Quantitative PCR

Real-time quantitative PCR was performed as described previously [6]. Primers and probe for TPM3 were ready-made reagents that recognized the low molecular weight isoforms 2 to 5 but not the high molecular weight isoform 1 (Pre-designed TaqMan Gene Expression Assay, Applied Biosystems, Foster City, CA). Primers and probe for GEP were GRN-forward (5′-CAA ATG GCC CAC CCA ACT GA-3′), GRN-reverse (5′-CCC TGA GAC GGT AAA GAT GCA-3′) and GRN-probe (5′-6FAM CCA CTG CTC TGC CGG CCA CTC MGBNFQ-3′) [6]. Primer and probe reagents for control 18S were ready-made reagents (Pre-designed TaqMan Assay Reagents, Applied Systems). The mRNA expressions of GEP and TPM3 were examined in 44 pairs of HCC tissues and their paralleled adjacent non-tumor liver tissues. The relative amount of GEP and TPM3 had been normalized with control 18S for RNA amount variation and calibrator for plate-to-plate variation. The mRNA expression was presented as the relative fold change.

### Immunohistochemical Staining

Protein expression of GEP and TPM3 were investigated by immunohistochemical staining on formalin-fixed and paraffin-embedded clinical specimens [6], [8]. Monoclonal anti-GEP antibody at a dilution of 1∶500 and polyclonal anti-TPM3 antibody at a dilution of 1∶100 was used in the staining. Immunohistochemistry was performed using the Dako Envision Plus System (Dako, Carpinteria, CA) following manufacturer’s instruction. Briefly, sections were deparaffinised with xylene and hydrated with ethanol and then distilled water. Antigen retrieval was performed by boiling in citrate buffer (pH 6) for 10 minutes. Endogenous peroxidase was inactivated followed by primary antibody incubation at room temperature and expression signal was detected by incubation with horseradish peroxidase-conjugated secondary antibody at room temperature. The brown stain was developed with diaminobenzidine as the chromogen and the section was counterstain with hematoxylin.

## Results

### Identification of TPM3 as a Predominant Binding Partner of GEP

To identify the GEP interacting proteins, co-immunoprecipitation was performed using the monoclonal anti-GEP antibody. Liver cancer cells Hep3B expressed a higher GEP levels compared to HepG2 cells, and both levels were significantly higher than normal liver tissues. In order to increase the detection efficiency by using GEP as the ligand, Hep3B cells transfected with GEP full-length construct (Hep3B FL) were used for overexpression of GEP protein. The predominant interacting partner was identified by separating and comparing the immunoprecipitated proteins of anti-GEP antibody and those of control setups (antibody alone and protein lysate alone) by one-dimensional SDS-PAGE. An obvious major differential band with the size at around 34 kDa was observed only in the reaction lane but not in the control reaction lanes (Figure 1). The major differential band was excised, in-gel digested with trypsin and analyzed by MALDI-TOF/TOF MS. Both peptide mass fingerprint (PMF) and MS/MS ion search analyses identified the major differential band as tropomyosin 3 (TPM3, also named tropomyosin alpha-3 chain and located at 1q21.2). The representative MS spectra and matched masses to peptides of corresponding protein were shown in Figure 1 and Table 1. Tandem MS analysis of a tryptic peptide at m/z 1284.8 revealed an amino acid sequence of KLVIIEGDLER, which was only present in all isoforms of TPM3, but not in any isoforms of TPM1, TPM2 and TPM4 (Figure 1D, Figure S1).

### Validation of the Interaction of GEP and TPM3

To confirm the results obtained from the co-immunoprecipitation and mass spectrometry, the interaction between GEP and TPM3 was validated by immunoprecipitation using anti-TPM3 antibody, then followed with immunoblot detection using anti-GEP antibody in Hep3B FL and HepG2 FL. A band with expected GEP size (90 kDa) was observed only on anti-TPM3 antibody-precipitated protein but not in the control reactions (Figure 2 A–B). The data indicated that GEP was immunoprecipitated by anti-TPM3 antibody and therefore verified the interaction of GEP and TPM3 proteins.

Expression of TPM3 was investigated on a panel of cell lines (Figure 2 C, Figures S2 and S3). TPM3 protein expression levels were shown to be correlated with the protein expression levels of GEP in which elevated TPM3 expression was observed in Hep3B FL and HepG2 FL cells (GEP overexpression), whereas decreased level of TPM3 expression was observed in Hep3B AS cells transfected with GEP-antisense fragment (GEP suppression) (Figure 2 C). Nonetheless, suppression of TPM3 by siRNA approach demonstrated insignificant effect on alterations of GEP mRNA and protein levels, and GEP expression modulations showed minimal effect on TPM3 mRNA levels (Figures S2 and S3, Text S1). Further investigation would be warranted to delineate the association between GEP and TPM3 protein levels.

### Correlation of GEP and TPM3 in Clinical Specimens

Real-time quantitative RT-PCR was performed to investigate the mRNA expression level of TPM3 in HCC and the paralleled adjacent non-tumorous liver tissues. A total of 44 pairs of HCC and non-tumor liver tissues were investigated. Elevated TPM3 levels were observed in tumor compared to non-tumor tissues (*P*  = 0.001) (Figure 3A). The expression levels of GEP and TPM3 were significantly correlated (n  = 88, Spearman’s ρ correlation coefficient  = 0.658, *P*<0.001) (Figure 3B). Increased GEP levels were associated with cancer recurrence [6], [7], thus the association of TPM3 with recurrence-free survival was examined. The patients were segregated into TPM3 high and low groups using the TPM3 median level as the cutoff value. The median recurrence-free survivals for TPM3-high and TPM3-low groups were 8.0 and 52.0 months, respectively. Patients with elevated TPM3 levels were shown to have poor recurrence-free survival (log-rank test, *P*  = 0.0496) (Figure 3C).

Protein expression of TPM3 was investigated by immunohistochemical staining, and TPM3 protein was observed only in tumor tissues but not in the non-tumorous tissues (Figure 4 A–D). The staining patterns of GEP and TPM3 were compared (Figure 4 E–H). Similar expression patterns were observed in the serial sections that stained for GEP and TPM3, and both proteins co-localized in the cytoplasm of the HCC cells.

## Discussion

Protein-protein interacting partners are traditionally identified by undertaking the Western blot approach with the use of specific antibodies. However, this type of approach is hypothesis-driven, and therefore requires a “good guess” of the potential interacting partners for choosing the specific antibodies to examine the protein-protein binding. The Western blot approach, hence, is usually not cost effective. Recent advances in proteomic technologies for identification of unknown proteins at low quantity have allowed us to use an unbiased and non-hypothesis driven approach to look for novel protein-protein interacting partners. In this study, we aim to identify the novel predominant interacting proteins of GEP in HCC. The first phase, identification phase, has been examined with co-immunoprecipitation using anti-GEP antibody to capture GEP and its interacting protein partners, followed with comparative 1D SDS-PAGE analysis and mass spectrometry analysis. Our result revealed an obvious differential protein band as the putative predominant GEP interaction partner, which was later shown to be TPM3. We subsequently confirmed their interactions and cellular co-localizations by Western blot analysis and histological examination in clinical specimens, respectively. Although we aimed to examine for the predominant binding partner of GEP in the present study, other minor binding partners of GEP may exist. Further studies for identifying the minor binding partners using nano-LC/MS method are on-going in our laboratories.

In the second phase of this study, validation phase, the interaction between GEP and TPM3 has been investigated with additional cell models, using anti-TPM3 antibody to capture TPM3 and its binding partners, followed by immunoblot to confirm the interaction of GEP with TPM3. Further investigation on clinical specimen demonstrated GEP and TPM3 to be overexpressed in HCC compared with non-tumor liver sample, and the expression levels of GEP and TPM3 were significantly correlated. Immunohistochemical staining also revealed that GEP and TPM3 are co-localised in the cytoplasm of HCC cells. The present study consolidated TPM3 as the interacting partner of GEP.

Tropomyosin is an actin-binding protein which exists in both muscle and non-muscle cells [16]. Function of tropomyosin has been well established in muscle cells in which it plays a central role in muscle contraction through regulating the cooperative binding of actin to myosin in response to the calcium ion flux. However, the role of tropomyosin remains unclear in non-muscle cells although its principle role is to stabilize and modulate the function of the actin filaments [17]. Tropomyosins belong to a multi-isoform family. There are approximately 40 isoforms of tropomyosin identified in mammals [18]–[19]. Distinct isoforms are believed to possess cell-type specific functions by binding to diverse actin filaments and thereby confer regulation of the microfilaments in different tissues [20]. hTM3 (a high molecular mass tropomyosin isoform, also referred as the tropomyosin alpha-1 chain isoform 4 [21]), but not hTM5 (a low molecular mass tropomyosin isoform, also referred as the TPM3 isoform 2 [22]), is involved in intracellular granule movements in the rat kidney epithelial cells [23]. In CHO cells, chimeric protein of hTM3 and hTM5 was involved in multinucleation and cytokinetic defects [24]. It would be important to differentiate the involvement of different isoforms. Nonetheless, the current proteomic approach identified the tryptic peptides (Figure 1) were conserved sequences common to TPM3 high and low molecular weight isoforms. Notably, the TPM3 antibodies (Figure 2 and 4) were raised against the TPM3 low molecular weight isoform 2. For TPM3 transcript quantification (Figure 3), the primer and probe set also recognized the low molecular weight isoforms 2 to 5. The liver cancer cells and clinical samples showed low/undetectable transcript levels of TPM3 high molecular weight isoform 1 (data not shown). Therefore, GEP should be associated with TPM3 low molecular weight isoforms in liver cancer. However, the association of GEP with TPM3 high molecular weight isoform in other cancer types could not be excluded because these isoforms have significant conserved sequences. Increased or decreased expression of different tropomyosin isoforms have also been reported in a number of human solid tumors [25]–[30], although the functional significance of differential expression is unclear.

In the present study, we have demonstrated elevated expression of TPM3 in HCC. Dysregulation of TPM3 has also been reported in other human diseases. Missense mutation in the TPM3 has been reported to be associated with autosomal nemaline myopathy, a disease characterized by the presence of muscle fibres in the pathognomonic rod bodies [31]–[34]. In anaplastic large-cell lymphoma, TPM3 is involved in hematopoietic tumorigenesis by forming TPM3*-*ALK (anaplastic lymphoma kinase) fusion through chromosome (1;2) translocation [35]. TPM3*-*ALK fusion gene is further investigated to be involved in transformation, proliferation, invasion and metastasis in anaplastic large-cell lymphoma [36]. Notably, chromosomal gain at 1q, 8q and 17q are frequently detected in HCC [25]. These chromosomal regions may contain important oncogenes or growth factors. TPM3 is located at the region of chromosome 1q21.2. In a recent study examining the HCC genetic aberrations using whole-genome array-CGH, TPM3 has been identified in the recurrent gain region on chromosome 1q as important for HCC tumorigenesis [25]. Therefore, overexpression of TPM3 would potentially be explained by gene amplification rather than mutation or gene fusion mechanism.

In summary, we are the first group to demonstrate that TPM3 is a predominant interacting partner of GEP in the cytoplasm of HCC cells. Notably, TPM3 has been reported to control migration, invasion and anchorage-independent growth of HCC cells [37], and previously we have reported that GEP regulates growth, invasion and anchorage-independent growth of HCC cells [6]. As the current study demonstrated TPM3 as the cytoplasmic interacting partner of GEP, thus the two molecules may act together to control the invasion and anchorage-independent growth ability of the HCC cells. Further studies to investigate other TPM family members with GEP on their potential protein-protein interactions would be warranted.

## Supporting Information

Figure S1 **Comparison of the reference protein sequences of TPM1-4.** Underlined regions were the MALDI-TOF/TOF-MS analysis of the tryptic peptides matched to TPM3. Mismatches were highlighted. The 5 isoforms of TPM3 are conserved in the tryptic peptide regions. The 7 isoforms of TPM1, the 2 isoforms of TPM2 and the 2 isoforms of TPM4 are conserved in the tryptic peptide regions as shown in the reference sequences.
(DOC)

Figure S2 **Suppression of TPM3 by siRNA.** Three different siRNAs against TPM3 were transfected to Hep3B and HepG2 cells respectively. The three controls included the parental cells only (c), cells incubated with lipofectamine only (lipo) and cells mock-transfected with siRNA negative control (NC). TPM3 suppression by siRNAs decreased the TMP3 mRNA and protein levels but showed insignificant effect on GEP levels.
(DOC)

Figure S3 **Modulation of GEP levels.** Suppression of GEP was performed in Hep3B cells with high endogenous GEP levels. Overexpression of GEP was performed in HepG2 cells with relatively low endogenous GEP expression. GEP expression modulations showed minimal effect on TPM3 mRNA levels.
(DOC)

Text S1 **Supplementary Materials and Methods.**
(DOC)
