# Supplementary material for: Identification and Characterization of Tropomyosin 3 Associated with Granulin-Epithelin Precursor in Human Hepatocellular Carcinoma
Source: PLoS One. 2012 Jul 6;7(7):e40324. doi: 10.1371/journal.pone.0040324 (PMC3391266; doi:10.1371/journal.pone.0040324)
Supplement: Figure S1 — Comparison of the reference protein sequences of TPM1-4. Underlined regions were the MALDI-TOF/TOF-MS analysis of the tryptic peptides matched to TPM3. Mismatches were highlighted. The 5 isoforms of TPM3 are conserved in the tryptic peptide regions. The 7 isoforms of TPM1, the 2 isoforms of TPM2 and the 2 isoforms of TPM4 are conserved in the tryptic peptide regions as shown in the reference sequences. (DOC) [file pone.0040324.s001.doc]

Figure S1

**TPM3 1** **MEAIKKKMQMLKLDKENALDRAEQAEAEQKQAEERSKQLEDELAAMQKKLKGTEDELDKY** **60**

TPM1 1 MDAIKKKMQMLKLDKENALDRAEQAEADKKAAEDRSKQLEDELVSLQKKLKGTEDELDKY 60

TPM2 1 MDAIKKKMQMLKLDKENAIDRAEQAEADKKQAEDRCKQLEEEQQALQKKLKGTEDEVEKY 60

TPM4 1 MEAIKKKMQMLKLDKENAIDRAEQAEADKKAAEDKCKQVEEELTHLQKKLKGTEDELDKY 60

**TPM3 61** **SEALKDAQEKLELAEKKAADAEAEVASLNRRIQLVEEELDRAQERLATALQKLEEAEKAA** **120**

TPM1 61 SEALKDAQEKLELAEKKATDAEADVASLNRRIQLVEEELDRAQERLATALQKLEEAEKAA 120

TPM2 61 SESVKEAQEKLEQAEKKATDAEADVASLNRRIQLVEEELDRAQERLATALQKLEEAEKAA 120

TPM4 61 SEDLKDAQEKLELTEKKASDAEGDVAALNRRIQLVEEELDRAQERLATALQKLEEAEKAA 120

**TPM3 121** **DESERGMKVIENRALKDEEKMELQEIQLKEAKHIAEEADRKYEEVARKLVIIEGDLERTE** **180**

TPM1 121 DESERGMKVIESRAQKDEEKMEIQEIQLKEAKHIAEDADRKYEEVARKLVIIESDLERAE 180

TPM2 121 DESERGMKVIENRAMKDEEKMELQEMQLKEAKHIAEDSDRKYEEVARKLVILEGELERSE 180

TPM4 121 DESERGMKVIENRAMKDEEKMEIQEMQLKEAKHIAEEADRKYEEVARKLVILEGELERAE 180

**TPM3 181**  **ERAELAESKCSELEEELKNVTNNLKSLEAQAEKYSQKEDKYEEEIKILTDKLKEAETRAE** **240**

TPM1 181 ERAELSEGKCAELEEELKTVTNNLKSLEAQAEKYSQKEDRYEEEIKVLSDKLKEAETRAE 240

TPM2 181 ERAEVAESKCGDLEEELKIVTNNLKSLEAQADKYSTKEDKYEEEIKLLEEKLKEAETRAE 240

TPM4 181 ERAEVSELKCGDLEEELKNVTNNLKSLEAASEKYSEKEDKYEEEIKLLSDKLKEAETRAE 240

**TPM3 241**  **FAERSVAKLEKTIDDLEDELYAQKLKYKAISEELDHALNDMTSI** **284**

TPM1 241 FAERSVTKLEKSIDDLEDELYAQKLKYKAISEELDHALNDMTSI 284

TPM2 241 FAERSVAKLEKTIDDLEDEVYAQKMKYKAISEELDNALNDITSL 284

TPM4 241 FAERTVAKLEKTIDDLEEKLAQAKEENVGLHQTLDQTLNELNCI 284
