# Supplementary figures and images for: Identification and Characterization of Tropomyosin 3 Associated with Granulin-Epithelin Precursor in Human Hepatocellular Carcinoma
Source: PLoS One. 2012 Jul 6;7(7):e40324. doi: 10.1371/journal.pone.0040324 (PMC3391266; doi:10.1371/journal.pone.0040324)

Figure S2

A Real-time quantitative RT-PCR

B Western Blot


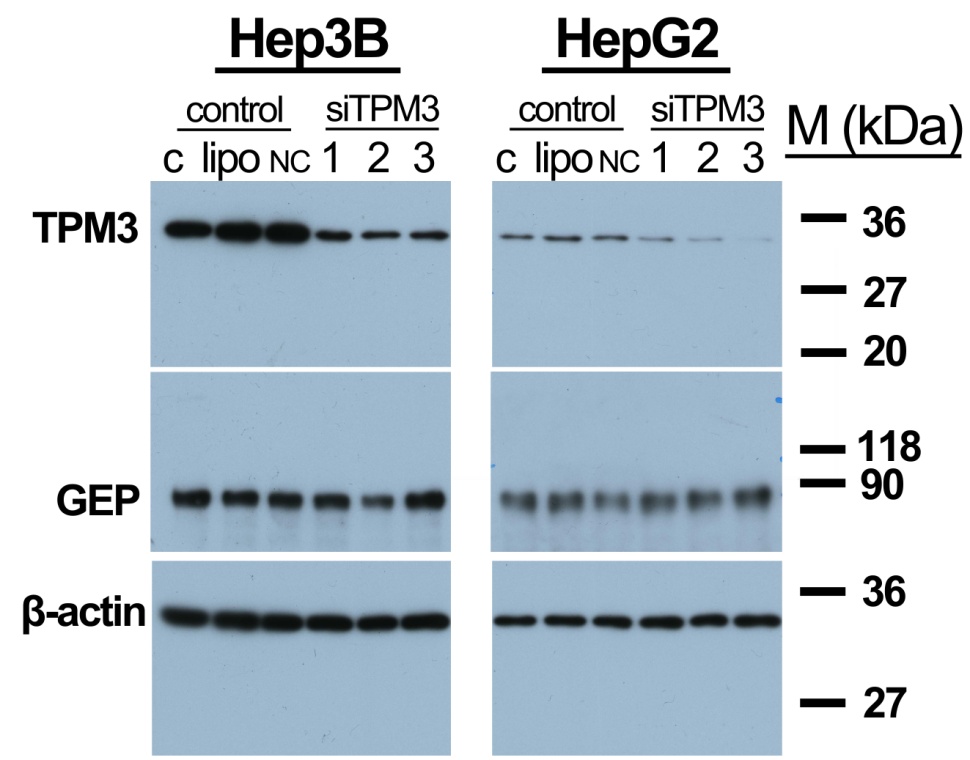

Supplement: Figure S2 — Suppression of TPM3 by siRNA. Three different siRNAs against TPM3 were transfected to Hep3B and HepG2 cells respectively. The three controls included the parental cells only (c), cells incubated with lipofectamine only (lipo) and cells mock-transfected with siRNA negative control (NC). TPM3 suppression by siRNAs decreased the TMP3 mRNA and protein levels but showed insignificant effect on GEP levels. (DOC) [file pone.0040324.s002.doc]
