# Supplementary material for: Identification and Characterization of Tropomyosin 3 Associated with Granulin-Epithelin Precursor in Human Hepatocellular Carcinoma
Source: PLoS One. 2012 Jul 6;7(7):e40324. doi: 10.1371/journal.pone.0040324 (PMC3391266; doi:10.1371/journal.pone.0040324)
Supplement: Text S1 — Supplementary Materials and Methods. (DOC) [file pone.0040324.s004.doc]

**Text S1. Supplementary Materials and Methods**

***Suppression of TPM3 by siRNAs***

The expressions of TPM3 in Hep3B and HepG2 cells were suppressed by transfection of siRNA targeted at all isoforms of TPM3 (Life Technologies). Reverse transfection using Lipofectamine^TM^ 2000 (Life Technologies) was adopted to introduce the siRNA. Briefly, 5 µg/ml lipofectamine and 300 nM siRNA were mixed in 1 ml AMEM in each well of a 6-well plate. After 15 minutes incubation, 2 ml of 1x10^5^ cells in complete AMEM were added to the transfection mixture in each well. Transfection was performed in parallel in 96-well plate where 60 µl of 3x10^3^ cells were added to 30 µl transfection mixture. Cells were then incubated at 37°C for 48 hours and harvested for Western blot (6-well) and qPCR (96-well) analysis.

***Western blot***

Cells were harvested by Trypsin-EDTA and incubated with cell lysis buffer (Cell Signaling) on ice for 20 minutes. Protein content was quantified by DC protein assay (Bio-Rad) and analyzed by SDS-PAGE. Antibodies against β-actin (Sigma), GEP monoclonal antibody (Ho JC *et al*., Hepatology 2008) and TPM3 antibody (Sigma) were used as primary antibody in the Western blot. Detection was then performed by horseradish peroxidase-labelled secondary antibodies with enhanced chemiluminescence.

***Real-time quantitative PCR***

The first strand cDNA was synthesized by the Cells-to-CT kit (Life Technologies) according to manufacturer’s instructions. The primers and probes used for TPM3, GEP and 18S qPCR assays have been described in the manuscript. The mRNA expression levels were presented as the relative fold change to the mean data of the controls.
